# Dose Reconstruction of Di(2-ethylhexyl) Phthalate Using a Simple Pharmacokinetic Model

**DOI:** 10.1289/ehp.1205182

**Published:** 2012-09-24

**Authors:** Matthew Lorber, Antonia M. Calafat

**Affiliations:** 1 Office of Research and Development, U.S. Environmental Protection Agency, Washington, DC, USA; 2 National Center for Environmental Health, Centers for Disease Control and Prevention, Atlanta, Georgia, USA

**Keywords:** DEHP, dose reconstruction, pharmacokinetic model, phthalate exposure

## Abstract

Background: Di(2-ethylhexyl) phthalate (DEHP), used primarily as a plasticizer for polyvinyl chloride, is found in a variety of products. Previous studies have quantified human exposure by back calculating intakes based on DEHP metabolite concentrations in urine and by determining concentrations of DEHP in exposure media (e.g., air, food, dust).

Objectives: To better understand the timing and extent of DEHP exposure, we used a simple pharmacokinetic model to “reconstruct” the DEHP dose responsible for the presence of DEHP metabolites in urine.

Methods: We analyzed urine samples from eight adults for four DEHP metabolites [mono(2-ethylhexyl) phthalate, mono(2-ethyl-5-hydroxyhexyl) phthalate, mono(2-ethyl-5-oxohexyl) phthalate, and mono(2-ethyl-5-carboxypentyl) phthalate]. Participants provided full volumes of all voids over 1 week and recorded the time of each void and information on diet, driving, and outdoor activities. Using a model previously calibrated on a single person self-dosed with DEHP in conjunction with the eight participants’ data, we used a simple trial-and-error method to determine times and doses of DEHP that resulted in a best fit of predicted and observed urinary concentrations of the metabolites.

Results: The average daily mean and median reconstructed DEHP doses were 10.9 and 5.0 µg/kg-day, respectively. The highest single modeled dose of 60 µg/kg occurred when one study participant reported consuming coffee and a bagel with egg and sausage that was purchased at a gas station. About two-thirds of all modeled intake events occurred near the time of reported food or beverage consumption. Twenty percent of the modeled DEHP exposure occurred between 2200 hours and 0500 hours.

Conclusions: Dose reconstruction using pharmacokinetic models—in conjunction with biomonitoring data, diary information, and other related data—can provide a powerful means to define timing, magnitude, and possible sources of exposure to a given contaminant.

Phthalates, diesters of phthalic acid, are a class of industrial chemicals extensively used as softeners of plastics, solvents in perfumes, and additives to many personal care and consumer products such as hairsprays, lubricants, and insect repellents (David et al. 2001; Koch and Calafat 2009). Di(2-ethylhexyl) phthalate (DEHP) is used primarily as a plasticizer for polyvinyl chloride and can be found in a variety of products, such as floor or wall coverings, vinyl gloves, toys and child care articles, materials that have contact with foods, and medical devices (Schettler 2006).

Several phthalates, including DEHP, di-*n*-butyl phthalate, diisobutyl phthalate, and butyl benzyl phthalate, have been identified as endocrine disruptors in animal studies (National Research Council 2008). In the body, phthalates rapidly hydrolyze to their respective monoesters; some monoesters are further metabolized by phase I and/or phase II reactions. For phthalates with short alkyl side chains, monoesters represent the major human metabolites. In contrast, for phthalates with longer alkyl chains, including DEHP, the main metabolites are the products of ω-, ω-1, and β-oxidations of the alkyl chain (Koch and Calafat 2009). All phthalate metabolites are excreted in the urine or feces within a few hours; excretion is complete within 1 or 2 days (Koch and Calafat 2009).

Two approaches have been used to quantify human exposures to DEHP and other phthalates. One relies on the biomonitoring of DEHP metabolites in urine to back calculate the daily intake of DEHP. For example, researchers have used biomonitoring data from the United States (Kohn et al. 2000; Lorber et al. 2010), Germany (Koch et al. 2003; Wittassek et al. 2007), and other countries (Fujimaki et al. 2006; Huang et al. 2006) to characterize general population exposures. The other approach uses DEHP concentrations in exposure media (e.g., air, food, dust) and exposure contact rates to estimate daily intakes. This approach has been used in two studies, one using worldwide exposure media data (Clark et al. 2011) and the other using European exposure data (Wormuth et al. 2006). Both approaches arrived at similar intakes of DEHP in adults, ranging from about 2 to 10 µg/kg-day. These researchers concluded that, for the general population, diet explained the bulk of exposure to DEHP.

For the present study, we used a third approach to assess general population exposures to DEHP. This approach relies on having precise times of urination and urine volumes, along with urinary concentrations of DEHP metabolites. We used a calibrated simple pharmacokinetic model for DEHP (Lorber et al. 2010) in conjunction with these data to “reconstruct” the dose necessary to have resulted in the observed metabolite concentrations.

## Methods

*Biomonitoring data set*. We used data collected from four adult men and four adult women, 24–59 years of age, who provided the full volume and time of all urinary void events for 1 week in October–November 2005. Each participant provided diary information that included when and what the participant ate and drank, time spent driving and putting gasoline in the car, and time spent in other activities that might influence the presence of DEHP metabolites and other contaminants in urine. Because we had no information about the participants’ body weights, we assumed body weights of 70 kg for men and 60 kg for women. The study design has been described in detail previously (Li et al. 2010; Preau et al. 2010; Ye et al. 2011). The institutional review board of the Centers for Disease Control and Prevention (CDC) approved the original study; the present study was exempted, and all participants provided written informed consent at the time of the original study.

The data set comprised 56 person-days of data (8 people × 7 days) and included 427 distinct urine samples. These samples have previously been analyzed for several polycyclic aromatic hydrocarbon (PAH) metabolites (Li et al. 2010), bisphenol A (Ye et al. 2011), monoethyl phthalate (a metabolite of diethyl phthalate), and two DEHP metabolites [mono(2-ethylhexyl) phthalate (MEHP) and mono(2-ethyl-5-hydroxyhexyl) phthalate (MEHHP)] (Preau et al. 2010). For the present study, we used the urinary concentrations of MEHP and MEHHP (Preau et al. 2010), as well as two other DEHP metabolites [mono(2-ethyl-5-oxohexyl) phthalate (MEOHP) and mono(2-ethyl-5-carboxypentyl) phthalate (MECPP)] that had been measured at the time of the original study but not reported by Preau et al. (2010). We also used times of urination, volume of urine voids, and diary information for each person.

*Dose reconstruction.* We used a DEHP model described previously (Lorber et al. 2010). This model is a simple two-compartment empirical model that was structured and calibrated based on the experimental data presented by Koch et al. (2005). The calibration (Lorber et al. 2010) provided all required model rate constants and other parameters. For the present study, the only required inputs were time and volume of each urine void. The dose reconstruction exercise entailed determining the time and extent of DEHP exposure that would lead to predicted concentrations matching the measured concentrations of DEHP metabolites. The structure of the model and its prior calibration are described in Supplemental Material, pp. 3–7 (http://dx.doi.org/10.1289/ehp.1205182).

To reconstruct the DEHP dose, we observed when urinary concentrations of DEHP metabolites increased and then assumed that this increase was related to a recent exposure event. By trial and error, we calibrated the extent and time of exposure to fit the observed urinary concentrations. The calibration of the model entailed visual inspection of the predicted concentrations compared with the observed concentrations. For example, if the MEHP concentration increased from approximately 10 μg/L to > 200 μg/L from one urination to the next (and the other metabolites also showed meaningful increases), we assumed that the rise was due to an exposure event that occurred after the first urination event but before the second one. By choosing different times of intake (i.e., exposure event times) and different intake quantities, we found an intake–time pair that resulted in what appeared to be the best fit of predicted and observed concentrations for all four metabolites; however, this fit may not have been optimal for all metabolites. A key assumption of the model is that DEHP exposure occurred as a bolus dose during a single time step of the model, which is consistent with an exposure via food consumption.

Three sensitivity analyses tests were performed. In one of these tests, we assumed that the DEHP dose was spread out over hours (i.e., 2, 8, and 24 hr) in 15-min time intervals around the calibrated time of exposure, rather than assuming exposure to a single bolus dose [see Supplemental Material, pp. 7–8 (http://dx.doi.org/10.1289/ehp.1205182)]. Specifically, we set half of the spread time before and half after the calibrated time of exposure. For the 24-hr simulations, we summed the total intakes for the day and assumed that exposure was evenly spread throughout the day; for the other two simulations, individual exposures were only spread out for the time periods evaluated (2 and 8 hr). In a second sensitivity test, we investigated timing of exposure by modeling a bolus dose occurring 2 hr before or 2 hr after the calibrated time of exposure. Last, we examined the final reconstructed daily dose and compared it to daily doses that might be determined from applying the creatinine-correction approach to individual spot samples [see Supplemental Material, pp. 9–12].

## Results

*Best fit to the data*. Using the rate constants from Lorber et al. (2010) for the eight study participants, we found a good fit to the data. This suggested validity of the calibrated parameters, which were determined experimentally based on one individual and then applied to the eight participants. However, a pattern observed in the original experimental data set used to calibrate the model (Koch et al. 2005) suggested a refinement to the best-fit solution in the current model application. Specifically, after approximately 24 hr, a small portion of the original dose of DEHP appeared to metabolize anew, in almost a second phase of metabolism. That is, the excretion of DEHP metabolites rose slightly on the second day in contrast to a continued first-order decline and loss of the metabolites (Lorber et al. 2010). We hypothesized that some of the spikes in urinary DEHP metabolite concentrations observed in our study population may have reflected a second phase of metabolism from earlier exposures rather than a unique new exposure, particularly when the metabolite concentrations were low and the calibrated dose was also small (near 1 µg/kg) [see Supplemental Material, pp. 5–7 (http://dx.doi.org/10.1289/ehp.1205182)]. Therefore, we removed small, calibrated exposures in a series of step-wise exclusions (Table 1). Removing modeled exposures < 1 µg/kg reduced the number of modeled intakes from 111 to 96 and increased the number of days with no exposures from 1 to 4, but had virtually no effect on the measures of central tendency (i.e., mean, median) compared with estimates from the simulation with all intakes. However, when all exposures < 2 µg/kg were removed, the number of days with no exposures rose to 14 and the median intake declined from 5.0 to 4.5 µg/kg-day. We observed a more significant impact when we removed all events resulting in exposures < 3 µg/kg. On the basis of these results, we removed all events with < 1 µg/kg for the final calibration. For additional details and analyses of secondary metabolism, see Supplemental Material, pp. 5–7 and Table S1.

**Table 1:** Comparison of model estimates from the initial baseline calibration (when all increases in urine DEHP metabolite concentrations were assumed to be the result of a unique exposure) with estimates generated after removing exposure events < 1, < 2, and < 3 µg/kg DEHP.

Description	Baseline	Baseline minus events < 1 µg/kg	Baseline minus events < 2 µg/kg	Baseline minus events < 3 µg/kg
No. of exposure events	111.0	96.0	71.0	56.0
Average median daily exposure (µg/kg‑day)	5.0	5.0	4.5	3.5
Average mean daily exposure (µg/kg‑day)	11.1	10.9	10.4	9.8
No. of person-days with no exposure	1.0	4.0	14.0	21.0

**Figure 1:**
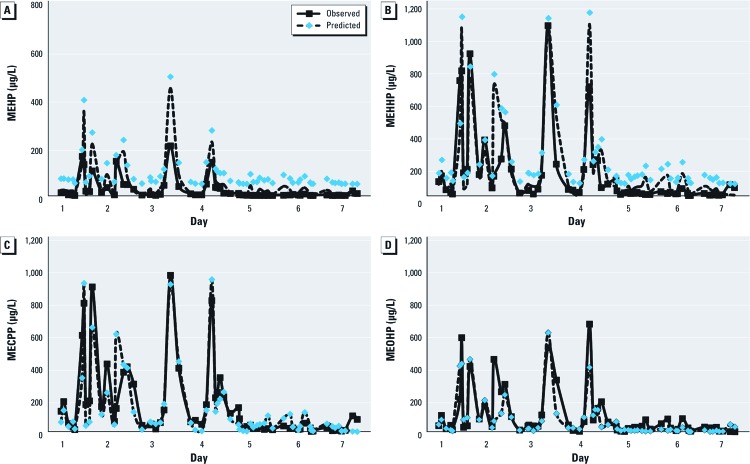
Observed and predicted urinary concentrations of the four DEHP metabolites for subject 1 throughout the study period. (*A*) MEHP. (*B*) MEHHP. (*C*) MECPP. (*D*) MEOHP. Corresponding figures for subjects 2–8 are available in Supplemental Material, Figures S1–S7 (http://dx.doi.org/10.1289/ehp.1205182).

*Characteristics of the best fit.* Figure 1 shows comparisons between predicted and observed urine concentrations of the four metabolites over a 7-day period for one participant [subject 1; for subjects 2–8, see Supplemental Material, Figures S3–S9 (http://dx.doi.org/10.1289/ehp.1205182)]. The model had a tendency to overpredict the urinary concentrations of MEHP, the hydrolytic monoester metabolite of DEHP (Figure 1A). MEHP has the most rapid elimination in humans, with a 5-hr half-life; MEHP also represents the smallest amount of the original DEHP dose (5.9%) (Koch et al. 2005). Therefore, because the secondary metabolites of DEHP are considered better indicators of exposure than MEHP (Barr et al. 2003), we chose to focus on finding the model with the best fit for the concentrations of MECPP, MEOHP, and MEHHP, at the expense of those of MEHP.

Although a visual inspection of Figure 1 suggests that trends in metabolite concentrations were well captured by the model, this may be, at least in part, a visual artifact. For example, the model seems to have predicted the observed high MEHHP concentration on day 3 (Figure 1B). However, the observed concentration of MEHHP for the next observation is 195 µg/L, whereas the model predicted 488 µg/L. For MECPP, the model predictions and observations are consistent for this time point, with both values near 400 µg/L (Figure 1C). However, for MEOHP, the observed value is higher than the prediction (320 µg/L and 112 µg/L, respectively; Figure 1D). This illustrates the trade-offs resulting from the use of the trial-and-error approach to find the best fit reconstructed dose for each individual over 7 days.

**Figure 2:**
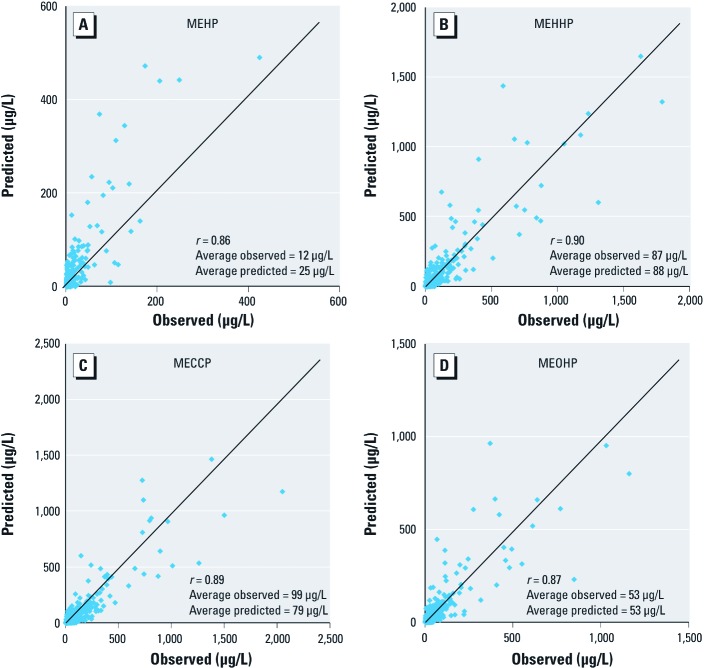
Correlations between observed and predicted urinary concentrations of the four DEHP metabolites for all 427 urination events for the eight study participants. (*A*) MEHP. (*B*) MEHHP. (*C*) MECPP. (*D*) MEOHP.

Figure 2 shows predicted versus observed concentrations of the four metabolites for all eight individuals and their 427 urination events. The correlations were excellent, with correlation coefficients (*r*) between 0.85 and 0.90. However, for MEHP, the model tended to overpredict concentrations (Figure 2A). Specifically, the average predicted concentration (25 μg/L) is just over twice the average observed concentration (12 μg/L). In contrast, the average predicted and observed concentrations of MEHHP (Figure 2B) are virtually identical (88 and 87 µg/L, respectively), with a similar spread of values above and below the best-fit line. The model has a slight tendency to underpredict MECPP concentrations, with an average predicted value of 79 µg/L, compared with an average observed value of 99 µg/L (Figure 2C). Results for MEOHP are similar to those of MEHHP: Predictions and observations are virtually identical with both averaging 53 μg/L, with a high correlation coefficient of 0.87.

*Characteristics of exposure.* The best-fit solution (baseline minus events with exposures < 1 µg/kg body weight; Table 1) contained 96 distinct exposure events, or about 1.7 events/person-day (96 events ÷ 56 person-days). Most person-days included 0, 1, or 2 exposure events per day, but there were 6 days with 3 events and 2 days with 4 events. The modeled mean and median daily DEHP exposures were 10.9 and 5.0 µg/kg-day, respectively. The daily mean intakes for the eight participants, from lowest to highest were 3.3, 4.3, 5.0, 9.8, 13.4, 15.4, 17.7, and 18.3 µg/kg-day. The distribution in selected exposure ranges for the 56 person-days of daily intakes is presented in Table 2. The highest single person-day of exposure was 80 µg/kg-day, and the second highest was 36 µg/kg-day.

**Table 2:** Distribution of exposure ranges for the 56 person-days of daily intakes.

Range (µg/kg‑day)	Person-days
0	4
1–5	21
6–10	13
10–20	8
> 20	10

**Figure 3:**
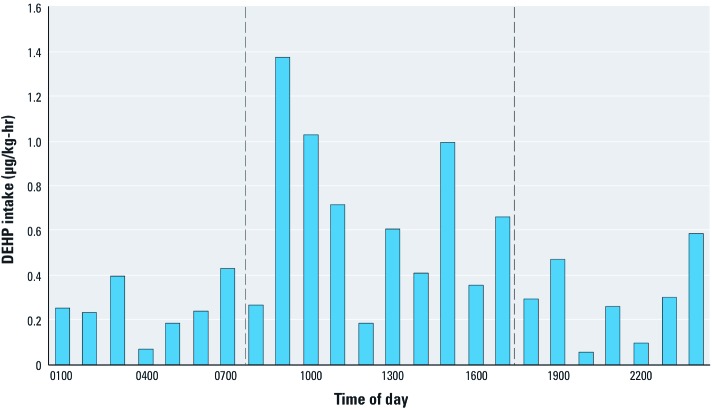
Average hourly model-based DEHP intakes for the eight study participants. Dotted lines highlight intakes that occurred between 0800 and 1700 hours (6.7 µg/kg‑day of the 10.9‑µg/kg‑day total).

Figure 3 shows the estimated average hourly DEHP intakes for the eight participants. The higher hourly averages for 0800–1700 hours suggest that about 60% of the DEHP intakes occurred when participants were away from home, although the study period did include 2 weekend days for which the exposures were more likely to occur at home.

We also superimposed the modeled time of exposure on participants’ diary information (data not shown). Of the 96 exposure events, 66 were predicted by the model to have occurred between 0500 and 2200 hours; of these, 42 were predicted to have occurred within 1 hr (before or after) of reported consumption of food and/or beverage. The single highest modeled exposure event, at 60 µg/kg, occurred at the same time that the participant reported consuming “bagel with egg, sausage, cheese, coffee bought at gas station.” We speculate that these food items might have been kept warm and stored in a setting and/or in contact with materials that contained DEHP and transferred it to the food.

**Figure 4:**
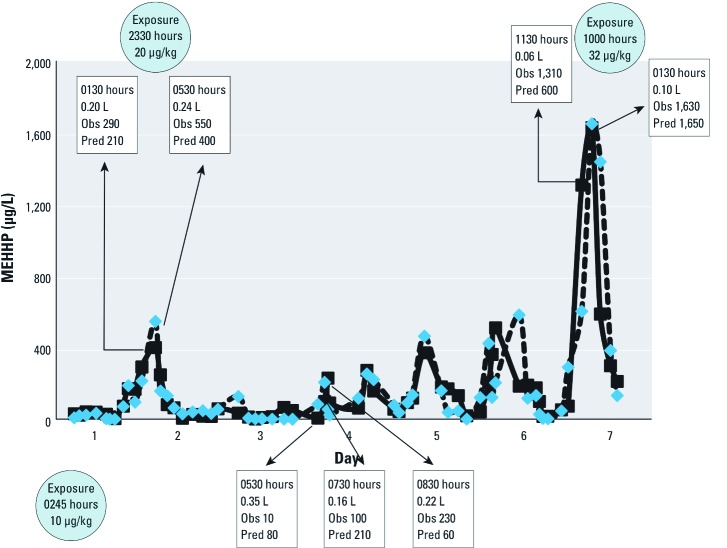
Observed (Obs) and predicted (Pred) urinary concentrations of MEHHP for the individual with highest nighttime DEHP intake (subject 3). Boxes indicate time of day, volume of urine, and MEHHP urinary concentrations (µg/L). Circles show time of day and magnitude of the calibrated exposure events.

Of 96 exposure events, 30 occurred at night (between 2200 and 0500 hours), and the average nighttime exposure was 2.2 µg/kg-day (about 20% of the daily total). None of the study participants reported food or beverage consumption at night during the study. However, model estimates indicated that one person with a relatively low average exposure (~ 3 µg/kg-day) had only nighttime exposures. In addition, the person with the highest average daily modeled DEHP exposure (18.3 µg/kg-day) experienced 41% of his/her exposure (~ 7.5 µg/kg-day on average) at night. Figure 4 shows examples of 2 nighttime exposure events and 1 daytime exposure event from this person. No reported activities in the person’s diary coincided with the nighttime exposure episodes at 2330 hours (20 µg/kg) and 0245 hours (10 µg/kg). The daytime exposure of 32 µg/kg, the second highest modeled exposure event in this study, occurred at 1000 hours on the seventh day of the experiment. No food or beverage consumption events were reported from 0900 to 1100 hours on that day, but the participant did report consumption of cereal at 0600 hours and tea at 1130 hours.

*Sensitivity analyses.* We ran additional simulations [see Supplemental Material, pp. 7–8 (http://dx.doi.org/10.1289/ehp.1205182)] that assumed the DEHP exposure events were spread out around the calibrated time point, in time periods of 2, 8, and 24 hr instead of 15 min. With 2-hr exposure events, the results were virtually identical (for example, see results for subject 3 in Supplemental Material, Figure S1). With exposure episodes of 8 hr, we observed a slight change in the location of the spikes in urinary concentrations of DEHP metabolites, but the trends remained similar. In contrast, for 24-hr exposure events, the predicted concentrations were discernibly different than the actual observations. The fact that the 2-hr spread resulted in virtually identical concentration predictions is expected from a modeling perspective, and possibly in reality. The model calculated void concentrations based on the mass of metabolite in the bladder at the time of the void event. The same mass would arrive in the bladder whether the modeled exposure was from small bolus doses spread out over a period of time prior to the void event or from one large bolus dose at a single time before the void event. However, because the large dose is spread into smaller and smaller bolus doses over a longer period of time, doses begin to overlap void events, so different amounts are delivered and available for each void, compared with the scenario of a single large bolus dose.

We conducted an additional sensitivity analysis to compare our model-based dose reconstruction with DEHP intakes calculated from creatinine-corrected metabolite concentrations in single-spot urine samples, as described in Supplemental Material (pp. 9–12; http://dx.doi.org/10.1289/ehp.1205182). Specifically, we estimated intakes based on the four individual metabolites and their sum for 62 individual urine samples collected from one study participant over the week of sampling, and for 42 individual urine samples collected between 0700 and 1900 hours each day for that study participant. The average model-based daily intake for this participant (18.3 µg/kg-day) was similar to the average daily intake calculated using the creatinine-correction approach (19.4 µg/kg-day) (see Supplemental Material, Table S2). However, daily predictions of intakes based on a single urine void using the creatinine-correction approach showed wide variability. For example, the average creatinine-predicted daily intake from nine void events on 1 day was 46.9 µg/kg-day based on the sum of the four metabolites, compared with the model-based reconstructed dose of 36.0 µg/kg-day [Thursday; see Supplemental Material, Table S2 (http://dx.doi.org/10.1289/ehp.1205182)]. The range in predictions over these nine events was very wide, however, at 4.0–99.5 µg/kg-day. We observed similar wide ranges in all days of the week, with the difference in high and low predictions ranging from a factor of 4 to a factor of 25. Further, if one considered only the void events during daytime hours [when an individual might logically contribute a sample to a survey program such as the National Health and Nutrition Examination Survey (NHANES)], the average predicted intake based on creatinine correction declined to 16.4 µg/kg-day (see Supplemental Material, Table S3). This finding of a difference between daytime and nighttime extrapolated intakes is similar to that found by Preau et al. (2010) for MEHHP metabolite concentrations in this cohort overall; they found that the geometric mean concentration of samples collected in the evening (33.2 µg/L) was significantly higher (*p* < 0.01) than in samples collected in the morning (18.7 µg/L) or in the afternoon (18.1 µg/L). At least for this individual and this cohort, urine samples taken during the day and used to extrapolate intakes using the creatinine-correction approach would result in an underestimate of overall intakes.

## Discussion

The model used in the present study appeared to accurately predict urine concentrations of DEHP metabolites in the eight adult volunteers who provided urine samples over a 7-day period. Our estimates of daily total exposures to DEHP were consistent with previous exposure estimates determined through other means for other populations. For example, the average DEHP intake measured in full-day diet samples consumed by a group of German adults was 2.4 µg/kg-day (Fromme et al. 2007). Clark et al. (2011) estimated median intakes of DEHP of 11.0 µg/kg-day, using worldwide data on concentrations of DEHP in exposure media in combination with contact rates. Wormuth et al. (2006) conducted a similar analysis, and their “intermediate” estimate of intake characterized as specific to Europe was 2.1 µg/kg-day (although like Clark, they used concentration data from around the world). Both Wormuth et al. and Clark et al. concluded that food was the primary exposure pathway for DEHP. Daily DEHP exposures have also been determined using surveys of DEHP metabolites in urine. Wittassek et al. (2007) estimated a median DEHP intake of 3.5 µg/kg-day using 24-hr urine samples from the German Environmental Specimen Bank for Human Tissues, collected from 634 German students between 1988 and 2003. Kohn et al. (2000) estimated a median intake of 0.7 µg/kg-day using MEHP urinary data from NHANES III, corresponding to years 1988–1994. Lorber et al. (2010) estimated a mean U.S. intake of DEHP in the range of 0.2–2.2 µg/kg-day based on DEHP metabolites data from NHANES 2001–2002.

The present analysis also suggested that DEHP exposure was dominated by the diet, as reported previously (Clark et al. 2011; Wormuth et al. 2006). However, for some individuals, significant DEHP exposures occurred at night, between 2200 and 0500 hours, when dietary exposures are unlikely. We used a modeling strategy to reconstruct DEHP exposures as single bolus events; possible nighttime bolus activities that could result in DEHP exposure include, for example, ingestion of water (or other beverage) and use of medications or personal care products. However, if the exposure had occurred over a number of hours instead of as a bolus dose as modeled, other possible exposures should be considered [e.g., inhalation exposures from use of a continuous positive airway pressure machine used for sleep apnea and/or snoring, oral exposure from a plastic mouth guard, emission of DEHP from vinyl materials (e.g., flooring) in the participant’s home]. Results of our sensitivity analysis suggested that a continuous inhalation exposure while the person was asleep could possibly explain the observed concentrations. In addition, moving the calibrated bolus intakes 2 hr before or 2 hr after the calibrated time did make a difference in the predictions, such that a calibrated time of exposure of 0200 hours does suggest, in fact, post-midnight exposure rather than pre-midnight exposure.

The tendency of our best-fit solution to overpredict MEHP might be explained by the use of experimental data from a male in his 60s (Koch et al. 2005) in the calibration of the model (Lorber et al. 2010). All eight participants in the present study were < 60 years of age, and seven of them were < 40 years of age. It could be that the older man excreted more MEHP than the younger persons because his body may not have been as efficient in quickly metabolizing MEHP as the younger individuals. Nonetheless, modeled concentrations for MEHP were the lowest of the four metabolites. The metabolism and excretion of the other three metabolites were well predicted with no change in calibrated rate parameters. Overall, we consider the model to be valid for this type of application.

About one-third of the person-days (18 of 56) had daily exposures ≥ 50% of the U.S. Environmental Protections Agency’s (EPA) reference dose (RfD) of 20 µg/kg-day (U.S. EPA 2012), and about 18% of the person-days had exposures above the RfD. The U.S. EPA (2012) defined the RfD as

an estimate (with uncertainty spanning perhaps an order of magnitude) of a daily exposure to the human population (including sensitive subgroups) that is likely to be without an appreciable risk of deleterious effects during a lifetime.

Although some participants in our study population—which is not representative of any specific population—experienced intakes at or near the RfD as estimated by the dose reconstruction, we cannot draw conclusions regarding the potential for health impact from such exposures to DEHP.

There is potential for using our approach to study exposure to chemicals ubiquitous in consumer products. We were fortunate to have a previously calibrated model and an experimental cohort with pertinent biomonitoring information (measurements in all collected urine samples) and diary information. Understandably, availability of this type of model and experimental data are rare. Using our model and data, we reconstructed DEHP exposures of eight individuals over 7 days, albeit with these provisos: *a*) Very small perturbations in urinary concentrations might not signal a unique exposure, but rather a second phase of metabolism from an earlier exposure; *b*) the data on which the model was calibrated showed higher urinary concentrations of MEHP than the experimental cohort (speculated to be the result of age in the individual whose data were used to originally calibrate the model); and *c*) it is possible that the exposure event that caused increases in DEHP metabolite concentrations in urine occurred over a period of time before the urinary event rather than as a bolus event.

Since the publication of the calibrated model (Lorber et al. 2010) used in this study, Anderson et al. (2011) established an extensive data set on human toxicokinetics of DEHP. In their study, 20 volunteers were dosed with stable-isotope–labeled DEHP and the urine was sampled and analyzed for DEHP metabolites over 48 hr. This is similar to the study on the single individual by Koch et al. (2005), which was used to calibrate the model used in the present study. Anderson et al. (2011) compared their data to Koch’s data and noted a similarly rapid elimination of metabolites, with most excreted within 24 hr. However, Anderson et al. (2011) noted that the four key metabolites of DEHP accounted for less of the dose than in Koch’s experiment (i.e., the molar fractions of excretion derived for each DEHP metabolite were lower than the molar fractions for each metabolite derived from Koch’s data). Anderson et al. (2011) discussed the implication of this difference in extrapolating results from a single spot urine sample to a daily exposure: Extrapolation factors would lead to higher daily estimated intakes compared with extrapolation factors based on Koch’s data. We would expect a similar impact if the model we used was recalibrated on Anderson’s data: that higher daily constructed doses would emerge.

Given these qualifications, some of the results were nonetheless compelling. For example, an individual’s reporting of consumption of food purchased at a gas station and a relatively high DEHP exposure calibrated to occur around the same time are unlikely to be a coincidence. The finding that certain individuals have meaningful nighttime DEHP exposures also warrants follow-up.

A key advantage of our approach is that it can reduce variability compared with the widely used creatinine-correction approach. We used a sensitivity analysis to compare our reconstructed daily intake estimate with estimates based on creatinine-corrected metabolite concentrations in individual urine samples taken during the day from a single participant. The reconstructed intake was 18.3 µg/kg-day, which was similar to the average intakes surmised by the creatinine-correction approach for nine urine events during the day (19.4 µg/kg-day). However, the range of creatinine-calculated intakes from the nine events was about 4–100 µg/kg-day.

Although it is data intensive, the modeling approach we advocate here has the potential not only to refine estimates of daily intakes and their variability but also to provide insight into potential pathways of exposure. In follow-up studies, a logical strategy would be to first screen a large number of individuals who have provided one urine sample to identify a smaller set of individuals that appear to have relatively high exposures. Then, this smaller set could be tracked over time with additional urine collections and comprehensive diaries to identify sources and pathways of exposure to DEHP. As for all phthalates, data on the DEHP content of consumer use products are limited (Kawakami et al. 2011). Several studies have concluded that DEHP exposure is dominated by food; although packaging and/or preparation with products containing DEHP has been proposed as the cause of food contamination, this has not been studied to any extent. Characterizing phthalate exposure through urine sampling is now and will likely remain the primary means to study total exposure, but studies that use detailed measurement data, diary data, and modeling can identify sources of exposure and possibly shed light on pathways not otherwise considered.

## Supplemental Material

(5.1 MB) PDF
