# Supplementary material for: Dose Reconstruction of Di(2-ethylhexyl) Phthalate Using a Simple Pharmacokinetic Model
Source: Environ Health Perspect. 2012 Sep 24;120(12):1705–10. doi: 10.1289/ehp.1205182 (PMC3548287; doi:10.1289/ehp.1205182)

Supplementary Material

Dose Reconstruction of Di(2-ethylhexyl) Phthalate Using a Simple Pharmacokinetic Model

Matthew Lorber[1], Antonia M. Calafat[2]

[1]Office of Research and Development, US EPA, 1200 Pennsylvania Ave, NW, Washington, DC, 20460. [2]National Center for Environmental Health, Centers for Disease Control, 1600 Clifton Rd, Atlanta, GA 30333

Table of Contents

A. Overview of Two-Compartment Pharmacokinetic Model

B. Analysis of Lower Metabolite Concentrations in Urine and the Possibility of Small Exposure Events

C. Sensitivity Analysis

D. Comparison of Reconstructed Dose with Dose Calculated Using Creatinine Correction

E. Complete Set of Calibrations for the 8 Individuals

References for Supplementary Section

Table S1. Ratio of MEHHP and MECPP urinary concentrations as a function of size of exposure

Table S2. Comparison of daily predictions of reconstructed dose with daily predictions of dose backcalculated using creatinine correction for participant S1, all urine events.

Table S3. Comparison of daily predictions of reconstructed dose with daily predictions of dose backcalculated using creatinine correction for participant S1, only urine events from 7 am to 7 pm.Figure S1. Sensitivity analysis for MEHHP, Subject 3, showing a comparison of the bolus dose assumption (a) with doses spread out over 2 h (b), 8 h (c), and 24 h (d)

Figure S2. Sensitivity analysis for MEHHP, Subject 3, showing a comparison of the base case dose assumption (a) with doses 2 h earlier (b) or 2 h later (c).

Figure S3. Observed and predicted urinary concentrations of the four DEHP metabolites, MEHP, MEHHP, MECPP, and MEOHP, for Subject 1.

Figure S4. Observed and predicted urinary concentrations of the four DEHP metabolites, MEHP, MEHHP, MECPP, and MEOHP, for Subject 2.

Figure S5. Observed and predicted urinary concentrations of the four DEHP metabolites, MEHP, MEHHP, MECPP, and MEOHP, for Subject 3.

Figure S6. Observed and predicted urinary concentrations of the four DEHP metabolites, MEHP, MEHHP, MECPP, and MEOHP, for Subject 4.

Figure S7. Observed and predicted urinary concentrations of the four DEHP metabolites, MEHP, MEHHP, MECPP, and MEOHP, for Subject 5.

Figure S8. Observed and predicted urinary concentrations of the four DEHP metabolites, MEHP, MEHHP, MECPP, and MEOHP, for Subject 6.

Figure S9. Observed and predicted urinary concentrations of the four DEHP metabolites, MEHP, MEHHP, MECPP, and MEOHP, for Subject 7.

Figure S10. Observed and predicted urinary concentrations of the four DEHP metabolites, MEHP, MEHHP, MECPP, and MEOHP, for Subject 8.

A. Overview of Two-Compartment Pharmacokinetic Model

The model used in this study is not a physiologically-based pharmacokinetic (PK) model, but rather an empirical PK model. As an empirical model, it is best understood as no more than a curve fit to the data. This has a few important implications for its general use. First, the structure and parameters of the model are not directly transferable to physiological measurements. The best example here is what is termed a "volume of distribution", or Vd, which is a common model parameter in both physiological and empirical PK models, but often a calibrated value and not a truly measured volume. It is generally used in terms of a blood volume, but the Vd when referring to blood is not equal to the amount of circulating blood at any time (which is about 5 L), but rather it is a volume of blood assigned to the model which results in a best fit of model predictions and blood measurements. It is most often equal to a volume larger than a physiological volume to reflect that a full intake, while assumed to deposit into this blood Vd, in reality deposits as well into other reservoirs of the body. The only way a model can duplicate the low concentrations in blood is to calibrate to a high blood Vd.

The first compartment of this two compartment empirical model is, in fact, a "blood" reservoir, in which DEHP metabolizes into several metabolites, four of which we studied for the present analysis. Then, the metabolites are transferred to a "bladder" reservoir where they await a urine void event to be fully eliminated. This empirical structure was based on the data in the controlled human dosing experiment described in Koch et al. (2005). An individual consumed a

known amount of labeled-DEHP and blood measurements were taken at 2 hr intervals up to 8 hours (4 measurements), and all urine voids (n=24) were fully collected for 48 hours. The model assumes 100% absorption into the blood reservoir. Measurements in the blood were of the DEHP metabolites and not DEHP. The calibrated Vd was 0.1 L/kg, or about 7 L for a 70 kg adult. Because the data are on phthalate metabolites, parent DEHP is assumed to convert to the metabolites while in the blood reservoir. Rate constants define the conversion of DEHP to metabolites, and further parameters define the transfer from the blood to the bladder reservoir. All metabolites present in the bladder reservoir at the time of a urine void are mixed into the volume of urine excreted during the void. Once the model was calibrated to data from the single individual, meaning that all pertinent rate constants were determined, the model can then be applied to other individuals. Input requirements for use with other individuals are simply the time and mass of DEHP exposure, and time and volume of urine voids.

Given the calibration to this study subject individual in Koch et al. (2005), one can expect that the model will predict an elimination of a user-supplied dose within the same time frame and with the same proportion of metabolite as found in the dosing experiment. The two important descriptors embedded in the two-compartment model rate parameters are known as the "molar fraction", termed Fue in the literature, and "elimination half-life", termed t ½ . The Fue is defined as the fraction of parent phthalate eliminated as metabolite, within a 24-hr time frame and on a molar basis, and the t ½ is defined as implied by the name, the time by which 50% of a given mass of metabolite will be eliminated in urine, derived as ln (2) divided by the rate constant of a first-order model fit to the urine data. The (Fue, t ½) for each metabolite found in the data and calibrated into the model were: MEHP – (0.059, 5 h), MEHHP – (0.233, 10 h), MEOHP (0.15, 10 h), and MECPP (0.185, 15 h).

An important concept for general use of the model on other individuals is that the internal rate constants should not need to be recalibrated. That is, for general use, one needs to accept that the toxicokinetics of DEHP in the controlled dosing experiment in one individual with one dose are appropriate for all people and all doses. If this is not the case, then the model has very limited usefulness. As described in the study, we found very good capabilities of the model on the eight individuals studied (with some caveats), suggesting these calibrated parameters and the model itself is useful in this type of application. The model is realized on an Excel © spreadsheet, with 15-min time intervals. Further details on the model can be found in Lorber et al. (2010).

B. Analysis of Lower Metabolite Concentrations in Urine and the Possibility of Small Exposure Events

Initially, the calibration targeted all circumstances where a rise in urinary concentrations of a given DEHP metabolite suggested a recent exposure event. The final calibration, however, removed the smallest exposure events, specifically all events less than 1 μg/kg. The justification for the removal of such exposures was that the increases in observed urinary concentrations of the phthalate metabolites may not have been due to unique exposures but rather to further metabolism of much earlier DEHP exposures, perhaps exposures than happened at least 24 hours before. This trend was observed in data from the self-dosed individual (Koch et al. 2005) from whom the model's rate constants were derived (Lorber et al. 2010), and in another experiment involving a group of college age persons who fasted for 48 h on bottled water only (Wittassek et al. 2011).

Lorber et al. (2011) discuss another trend pertinent to this discussion. They note that, after exposure to DEHP, excretion of the secondary metabolite, MECPP, is initially less than that of another secondary metabolite, MEHHP. However, over time, particularly into the second day post exposure, the MECPP concentration is higher than that of MEHHP. This trend is seen in the original data used to calibrate the model (Koch et al., 2005) as well as in a more recently published experimental dosing study (Anderson et al. 2011). Lorber et al. (2011) studied this trend using NHANES data and the implications for extrapolating daily dose from spot samples. Specifically, they found that the ratio of these two DEHP metabolites concentrations was close to 1.0 near the time of exposure, but ranged from above 1.5 up to 2.0 24 h after exposure. For the current study, we evaluated whether such a trend could be found in the data from the eight individuals in the experimental cohort. We hypothesized that the small perturbations in DEHP metabolite urinary concentrations which occurred when the concentrations were low to start with were not the result of an exposure, but rather the result of further metabolism of earlier DEHP exposures. If this were the case, than the ratio of MECPP to MEHHP concentrations would be close to the 1.5 to 2.0 range during these small perturbations.

We constructed Table S1 using the experimental data on the 8-person cohort. In the "original calibration," we identified all possible exposure events regardless of their magnitude. Using this original calibration, we determined the MECPP/MEHHP concentration ratio near calibrated exposure events that were less than 1 μg/kg, for events greater than or equal to 1 but less than 2 μg/kg, and events greater than 10 μg/kg. We noted the concentrations of these two metabolites and their ratio in the two urine events that followed the calibrated dose events. At times, we used only one urine event in our analysis because another calibrated dose occurred before the second urine event after the small exposure event. For example, there were 15 events

less than 1 μg/kg in the original calibration and 25 individual urine events following these small calibrated exposures.

The final results of this analysis are shown in Table S1. We found that the average MECPP/MEHHP concentration ratio associated with exposure events less than or equal to 1 μg/kg was 1.66, suggesting that the presence of these metabolites in urine resulted from earlier exposures Also, the metabolite concentrations near these small calibrated exposures were relatively low. In contrast, the average ratio when the perturbations and model calibrations suggested larger exposure events, above 10 μg/kg, was 1.13, and with much higher metabolite urinary concentrations. The middle tested range of exposures, between 1 and 2 μg/kg, appear to be a mix (some events possibly due to actual recent exposure with some likely not due to a recent exposure) with a ratio of 1.36.

In conclusion, this ratio analysis supports our strategy to remove small calibrated events. The rise in urine concentrations of the metabolites when the concentrations are low to start with appear to be likely a result of the second phase of metabolism of earlier exposures.

C. Sensitivity Analysis

We evaluated two issues in the calibration strategy with a simple sensitivity analysis exercise. One issue is the assumption that exposures were bolus doses. The second issue is the timing of the exposure. We conducted both sensitivity analyses using the calibration of one of the study participants and visually examining the impact on the graph showing predicted and observed urinary concentrations of MEHHP over time.

In the first exercise, the central time point of the calibrated exposure was the same, but we spread the total mass of the exposure in 2 h, 8 h, and 24 h around the calibrated time. For the 2 h analysis, we divided the total dose into eight 15-min increments and had four segments preceding and four segments following the calibrated time. Similarly, for the 8 h sensitivity test, we divided the dose into 32 15-min segments (4 hours or 16 segments before and 4 hours after the calibrated dose time). For the 24 h sensitivity test, we summed all of the exposure events and spread it out over 96 15-min increments. Figure S1 shows the bolus dose calibration and the 3 tested time increments for MEHHP for participant 3. Of interest, the predictions that result from the 2 h dose spread appear virtually identical to the bolus dose assumption, as seen by the similarity in Figures S1a and S1b. The reasons for this similarity are described in the manuscript. However, small differences can be found. For example, the highest concentration for this individual, over 1600 μg/L on day 7, is better predicted with the bolus dose calibration, Figure S1a, as compared with the 2 h spread, Figure S2b, and the event after this high concentration is lower for the bolus dose calibration as compared to the 2 hr spread. By contrast, more clear differences between predicted and observed MEHHP urinary concentrations are seen for the 8 h sensitivity test, particularly around this same high concentration on day 7. Similarly, for the 24 h sensitivity test, predicted and observed concentrations generally rise and fall concurrently, likely due to the generally higher daily exposures, but the match between predicted and observed concentrations is clearly not as good as with the other assumptions.

Figure S2 shows the results of the second sensitivity test for MEHHP for the same individual (participant 3). The match between predicted and observed MEHHP concentrations when moving the calibrated time either 2 hours before or 2 hours after the calibrated dose wasn't as good as the match obtained with the calibrated time.

D. Comparison of Reconstructed Dose with Dose Calculated Using Creatinine Correction

A common approach to calculating daily intakes of phthalates is the creatinine correction approach. This approach uses data from a spot sample including creatinine mass excreted, phthalate metabolite mass excreted, and other parameters to estimate a daily intake. Several references describe the derivation of this approach and its application to phthalates; no details will be provided here (Mage et al., 2008;Wittassek et al., 2011; Fromme et al., 2007). Very briefly, equations have been developed to estimate an individual's total daily excretion of muscle creatinine based on age, weight, height, sex, and race. Most surveys using spot urine samples now include concentrations of creatinine along with those of contaminants. The ratio of the spot sample creatinine excretion to total daily excretion is assumed to be equal to the ratio of the spot sample metabolite excretion to total daily metabolite excretion. In this construct, the unknown is the total daily metabolite excretion, which is then easily calculated. This equality assumes that both creatinine and the metabolite are excreted at relatively constant rates over the course of a day, and whereas that may be reasonable for muscle creatinine, it is at best a simplistic assumption for phthalate metabolite. Given the toxicokinetics of creation and excretion of phthalate metabolites, with elimination half-lives on the order of hours, metabolite excretions are highest near the time of exposure and decline exponentially thereafter. A second assumption made for use of this backcalculation approach is that exposure is constant and essentially daily such that estimated daily excretions of parent phthalate (calculated from daily excretion of phthalate metabolite mass corrected to parent phthalate mass using molar fractions and molecular weights) will equal daily intake of parent phthalate. Nearly 100% frequency of occurrence of

phthalate metabolites in surveys (such as NHANES) lends some credence to this assumption, but still it should be obvious that there will be large variability to contend with when using the creatinine correction approach for calculating daily phthalate intakes.

An exercise was conducted to compare the reconstructed daily dose of DEHP in this paper with DEHP intakes calculated from single spot events and the five metabolites. The exercise was conducted for 1 of the 8 participants – participant number 1 – who had 62 void events over 7 days and had the highest average daily reconstructed intake in this study population at 18.3 µg/kg-day. The creatinine correction approach was applied to each of four phthalates individually, and to the sum of the four phthalates. The results of this exercise are shown in Tables S2 and S3. Table S2 derives these values for all void events in the 24-hr day, and Table S3 only looks at void events which between the hours of 7 am and 7 pm. These times were chosen to correspond roughly to when it might be realistic for an individual to come into a center to supply a void for a survey such as NHANES. Creatinine-corrected intake results in these tables include, for each day and metabolite: the number of void events, the average backcalculated intake over the day, the standard deviation of that average, and the range of intakes. In each table, the creatinine-based intakes were compared with the modeled reconstructed dose

Several observations are made on these results:

1. **There is a difference in creatinine-based intakes between the metabolites.** Intakes calculated based on MEHP were the lowest and intakes based on MECCP were the highest. Different creatinine-based intake calculations for different DEHP metabolites has been discussed elsewhere (Koch et al, 2003) and will not be discussed further here. It is judged that the best

approach relies on the sum of all four metabolites and it is seen from the tables that the intakes calculated using the sum are bracketed within the intakes calculated by any of the individual metabolites.

2. **The general trends and overall average intake for this individual are comparable for both methods.** Focusing on Table S2 and the results based on the sum of the four metabolites, it is seen that the overall average daily intakes are very similar at 19.4 and 18.3 µg/kg-day for the creatinine-corrected and dose-reconstructed methods, respectively. With some reasonable and not unexpected differences, the first four days of the week were modeled to have high exposures at between 16 to possibly as high as 47 µg/kg-day by both methods, with the last three days modeled both ways to be under 10 µg/kg-day.

3. **There is a very wide range of variability for intakes calculated by individual spot urine samples using the creatinine-correction method.** This is perhaps the most revealing and interesting trend in this exercise. Looking at Table S2, intakes ranged well over an order of magnitude for almost every day, with ranges like 4.7 to 90.4 µg/kg-day.

4. **A closer look at the results suggests that this variability is due to much higher predictions of intake based on urine voids occurring during the evening.** Table S3 summarizes the results from over half of all urine events, those which occur during the day between 7 am and 7 pm. It is surmised that these would be reasonable times that individuals would supply a urine sample for a survey such as NHANES. However, the metabolite concentration in urine is more keyed to when the individual was exposed than to when he or she provides a sample for a survey. The highest intakes for this individual occurred on Monday and Thursday. On Monday, the total intake was reconstructed to be 26 µg/kg-day, with 9 µg/kg-day

predicted to occur at 2:15 pm and 10 µg/kg-day to occur at 8:30 pm. The remainder for that day, 7 µg/kg-day, was predicted to occur before 10 am. With this afternoon and evening exposure, urine concentrations would be highest at night, with high creatinine-predicted intakes ranging from 44 to 90 µg/kg-day in urine events occurring at 6 pm, 7 pm, 9 pm, 10 pm, and 11:30 pm. When not including the nighttime intake estimations for 9 pm, 10 pm, and 11:30 pm, the daily average for the creatinine approach declines from 36.8 (table S2, all four-based) to 27.7 (Table S3, all four-based), Similar, though smaller, declines are seen from Tuesday, Wednesday, Thursday, Friday, and Sunday, though a slightly higher average is seen for Saturday when looking only at daytime extrapolations. Overall, the daily average extrapolations for creatinine are fairly similar when considering the daytime hours only in comparison to all hours. However, a different story emerges if looking at creatinine extrapolations only from 7 am to 5 pm instead of from 7 am to 7 pm. There, the average intake for 7 days drops to 9.6 µg/kg-day, and on Monday, the average intake declines to 5.5 µg/kg-day since the 5 voids from 6 pm on are not included in the average. This finding of a difference between daytime and nighttime extrapolated intakes is similar to what Preau et al (2010) found for MEHHP metabolite concentrations in this cohort overall; they found that the geometric mean concentration of samples collected in the evening, 33.2 µ µg/L, was significantly higher ($p < 0.01$) than in samples collected in the morning, 18.7 µ µg/L, or in the afternoon, 18.1 µ g/L.

For this individual, the creatinine approach may have slightly underestimated his/her intake had samples only been provided during the day and not at night. It is not clear that this is a trend for the general public – for some people, just the opposite could emerge. But what is likely to be true for all individuals is that the variability in the predicted daily intakes using the creatinine correction approach will be high if considering all urine events during the day.

E. Complete Set of Calibrations for the 8 Individuals

Figures S3 through S9 include the modeled and observed results for the four metabolites for study participants 2 through 8 of the experimental cohort; results for study participant 1 is provided as Figure 3 in the main article.

References for Supplementary Section

Anderson WAC, Castle L, Hird S, Jeffery J, Scotter MJ. 2011. A twenty-volunteer study using deuterium labelling to determine the kinetics and fractional excretion of primary and secondary urinary metabolites of di-2-ethylhexylphthalate and di-iso-nonylphthalate. Food and Chem Tox. 49: 2022-2029.

Fromme H, Gruber L, Schlummer M, Wolz G, Bohmer S, Angerer J, et al. 2007. Intake of phthalates and di(2-ethylhexyl)adipate: Results of the Integrated Exposure Assessment Survey based on duplicate diet samples and biomonitoring data. Environ Int 33:1012–20.

Koch HM, Drexler H, Angerer J. 2003. An estimation of the daily intake of di(2-ethylhexyl)phthalate (DEHP) and other phthalates in the general population. Int. J. Hyg Environ Health 206: 77-83.

Lorber M., Koch HM, Angerer J. 2011. A critical evaluation of the creatinine correction approach: Can it underestimate intakes of phthalates? A case study with di-2-ethylhexyl phthalate. J. Expo. Sci. Environ. Epidemiol. 21: 576–586.

Mage DT, Allen RA, Kodalia A. 2008. Creatinine corrections for estimating children's and adult's pesticide intake doses in equilibrium with urinary pesticide and creatinine concentrations. J. Exp Sci Env Epi 18: 360–368.

Preau JL, Wong LY, Silva MJ, Needham LL, Calafat AM. 2010. Variability Over 1 Week in the Urinary Concentrations of Metabolites of Diethyl Phthalate and Di(2-Ethylhexyl) Phthalate Among Eight Adults: An Observational Study. Env Health Persp. 118:1748-54.

Wittassek M, Koch HM, Angerer J, Bruning T. 2011. Assessing exposure to phthalates – The human biomonitoring approach. Mol. Nutr. Food Res. 55: 7–31.

Table S1. Ratio of MEHHP and MECPP urinary concentrations as a function of size of exposure

Description	Exposure events of “Original Calibration”		
	< 1 µg/kg	1 - < 2 µg/kg	> 10 µg/kg
Number of exposure events	15	25	22
Number of urine events	24	50	41
MEHHP average concentration (µg/L)	14	24	373
MECPP average concentration (µg/L)	19	31	375
MECPP/MEHHP average concentration ratio	1.66	1.36	1.13

Table S2. Comparison of daily predictions of reconstructed dose with daily predictions of dose backcalculated using creatinine correction for participant S1, all urine events.

Day	Creatinine Correction Intakes, µg/kg-day: Mean / SD; Range					Reconstructed Dose µg/kg-day
	MEHP-based	MEHHP-based	MEOHP-based	MECCP-based	All Four-based	
Mon (n=9)	22.0<br>22.3; 2.0-60.7	35.3<br>32.3; 4.2-87.8	29.7<br>27.1; 3.6-74.5	48.4<br>43.0; 6.5-113.8	36.8<br>33.2; 4.7-90.4	26.0
Tue (n=7)	9.0<br>4.3; 3.8-14.9	17.8<br>9.3; 8.8-30.9	15.2<br>8.1; 7.5-27.2	27.8<br>13.2-45.7	19.4<br>8.8; 9.4-32.9	13.0
Wed (n=8)	11.8<br>14.9; 2.6-45.1	14.2<br>17.7; 4.1-55.5	12.8<br>15.9; 3.8-50.2	22.8<br>22.6; 7.8-64.2	16.3<br>18.2; 5.3-56.2	33.0
Thu (n=9)	30.2<br>26.4; 2.0-72.2	40.7<br>37.5; 2.7-94.3	37.2<br>33.7; 3.0-85.9	66.9<br>51.8; 7.1-129.9	46.9<br>39.5; 4.0-99.5	36.0
Fri (n=11)	2.7<br>3.1; 0.3-10.9	6.0<br>5.9; 0.9-22.0	5.7<br>5.8; 0.9-21.3	12.5<br>11.6; 2.9-44.1	7.6<br>7.3; 1.4-27.5	8.0
Sat (n=10)	2.2<br>1.3; 0.6-4.4	2.7<br>1.3; 0.7-4.7	2.6<br>1.4; 0.5-4.4	5.0<br>1.6; 2.5-7.1	3.4<br>1.3; 1.6-5.2	8.0
Sun (n=8)	3.9<br>5.8; 0.4-15.0	4.3<br>5.7; 0.9-14.9	4.2<br>5.6; 0.8-14.1	8.1<br>10.8; 1.8-28.9	5.4<br>7.2; 1.1-18.6	4.0
**Overall Average** *	**11.7**	**17.3**	**15.3**	**27.4**	**19.4**	**18.3**

* Overall Average is calculated as the average of daily averages.

Table S3. Comparison of daily predictions of reconstructed dose with daily predictions of dose backcalculated using creatinine correction for participant S1, only urine events from 7 am to 7 pm.

Day	Creatinine Correction Intakes, µg/kg-day: Mean / SD; Range					Reconstructed Dose µg/kg-day
	MEHP-based	MEHHP-based	MEOHP-based	MECCP-based	All Four-based	
Mon (n=6)	20.3<br>28.0; 2.0-60.7	26.9<br>35.8; 4.2-87.8	23.4<br>30.7; 3.6-74.5	34.0<br>43.8; 6.5-113.8	27.7<br>36.3; 4.7–90.4	26.0
Tue (n=5)	7.8<br>2.8; 3.8-9.6	16.3<br>4.1; 8.8-18.4	13.6<br>3.8; 7.5-16.2	24.0<br>6.3; 13.2-28.3	17.2<br>4.4; 9.4-20.0	13.0
Wed (n=6)	12.5<br>17.0; 2.6-45.1	14.8<br>20.2; 4.4-55.5	13.3<br>18.2; 4.2-50.2	19.5<br>22.2; 7.8-64.2	15.7<br>20.1; 5.3-56.2	33.0
Thu (n=5)	32.8<br>31.1; 2.0-72.2	38.7<br>39.7; 2.7-94.3	33.7<br>33.3; 3.0-77.8	56.8<br>54.2; 7.1-129.9	42.6<br>41.8; 4.0-99.5	36.0
Fri (n=8)	2.3<br>1.5; 0.8-5.0	4.9<br>2.8; 3.0-11.8	4.7<br>3.0; 2.8-11.8	10.5<br>5.1; 7.5-22.9	6.3<br>3.4; 4.9-14.5	8.0
Sat (n=6)	2.5<br>1.5; 0.6-4.4	3.2<br>1.3; 1.3-4.7	3.1<br>1.3; 1.2-4.4	5.1<br>1.8; 2.5-7.1	3.7<br>1.4; 1.6-5.2	8.0
Sun (n=6)	0.8<br>0.3; 0.4-1.2	1.2<br>0.4; 0.9-2.0	1.1<br>0.4; 0.8-2.0	2.4<br>0.7; 1.8-3.6	1.5<br>0.5; 1.1-2.4	4.0
**Overall Average***	**11.3**	**15.1**	**13.3**	**21.8**	**16.4**	**18.3**

- Overall Average is calculated as the average of daily averages.

Figure S1. Sensitivity analysis for MEHHP, Subject 3, showing a comparison of the bolus dose assumption (a) with doses spread out over 2 h (b), 8 h (c), and 24 h (d).

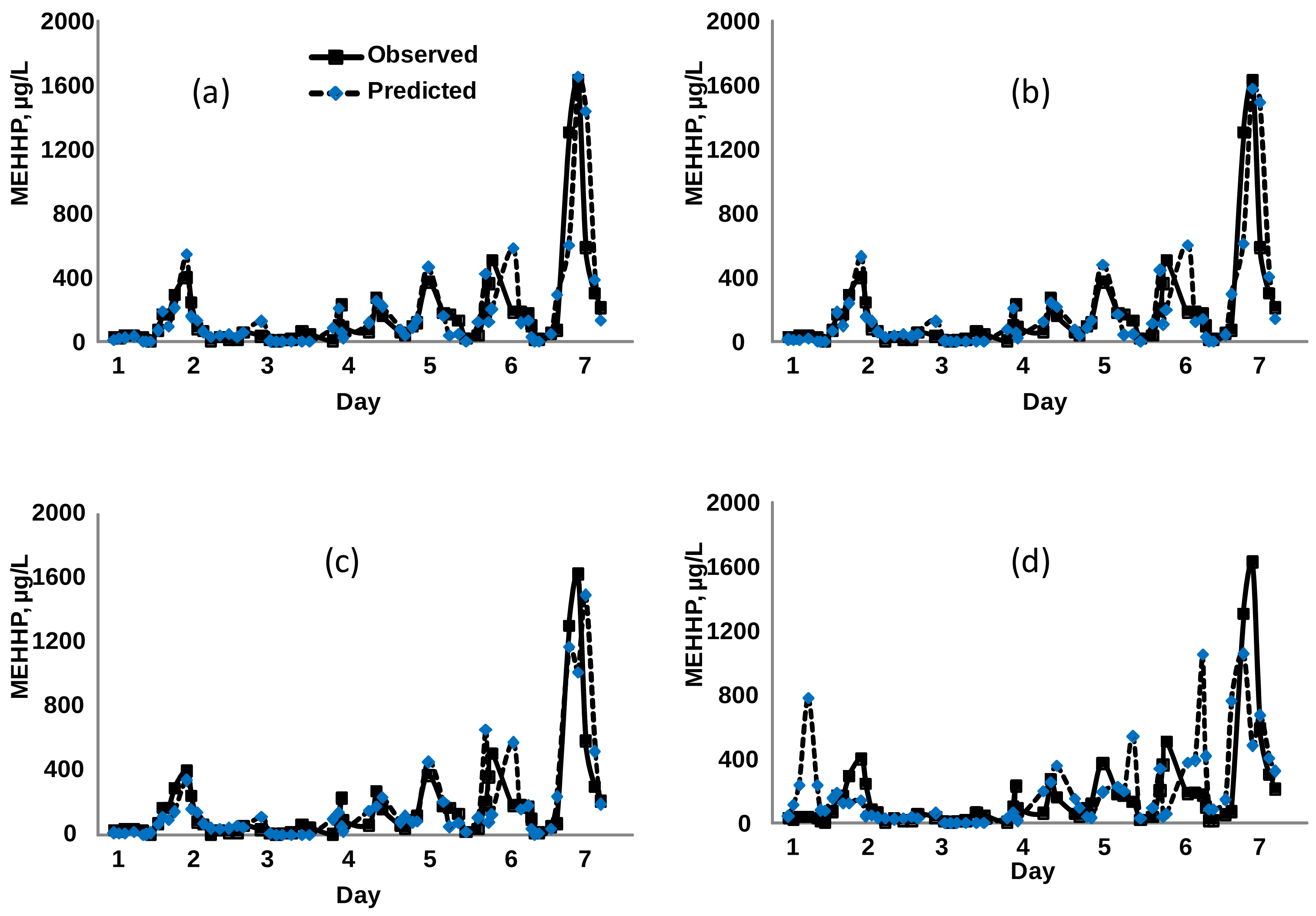

Figure S2. Sensitivity analysis for MEHHP, Subject 3, showing a comparison of the base case dose assumption (a) with doses 2 h earlier (b) or 2 h later (c).

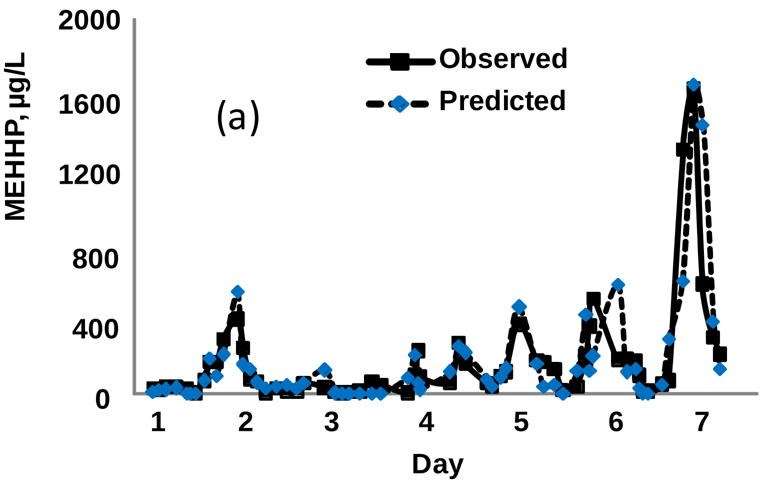

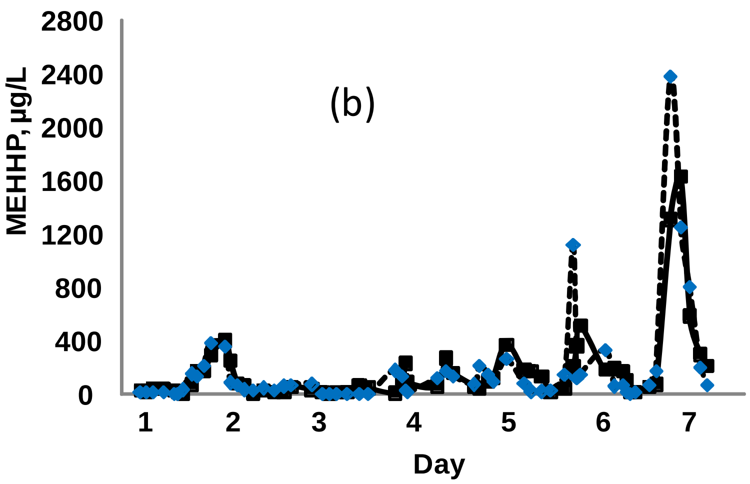

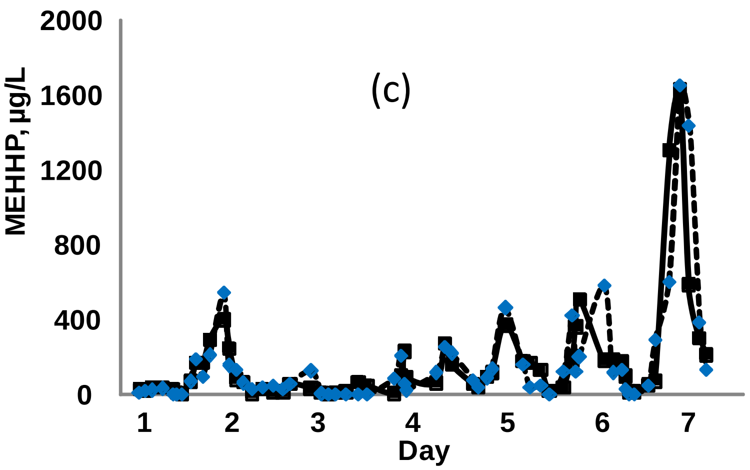

Figure S3. Observed and predicted urinary concentrations of the four DEHP metabolites, MEHP, MEHHP, MECPP, and MEOHP, for Subject 2.

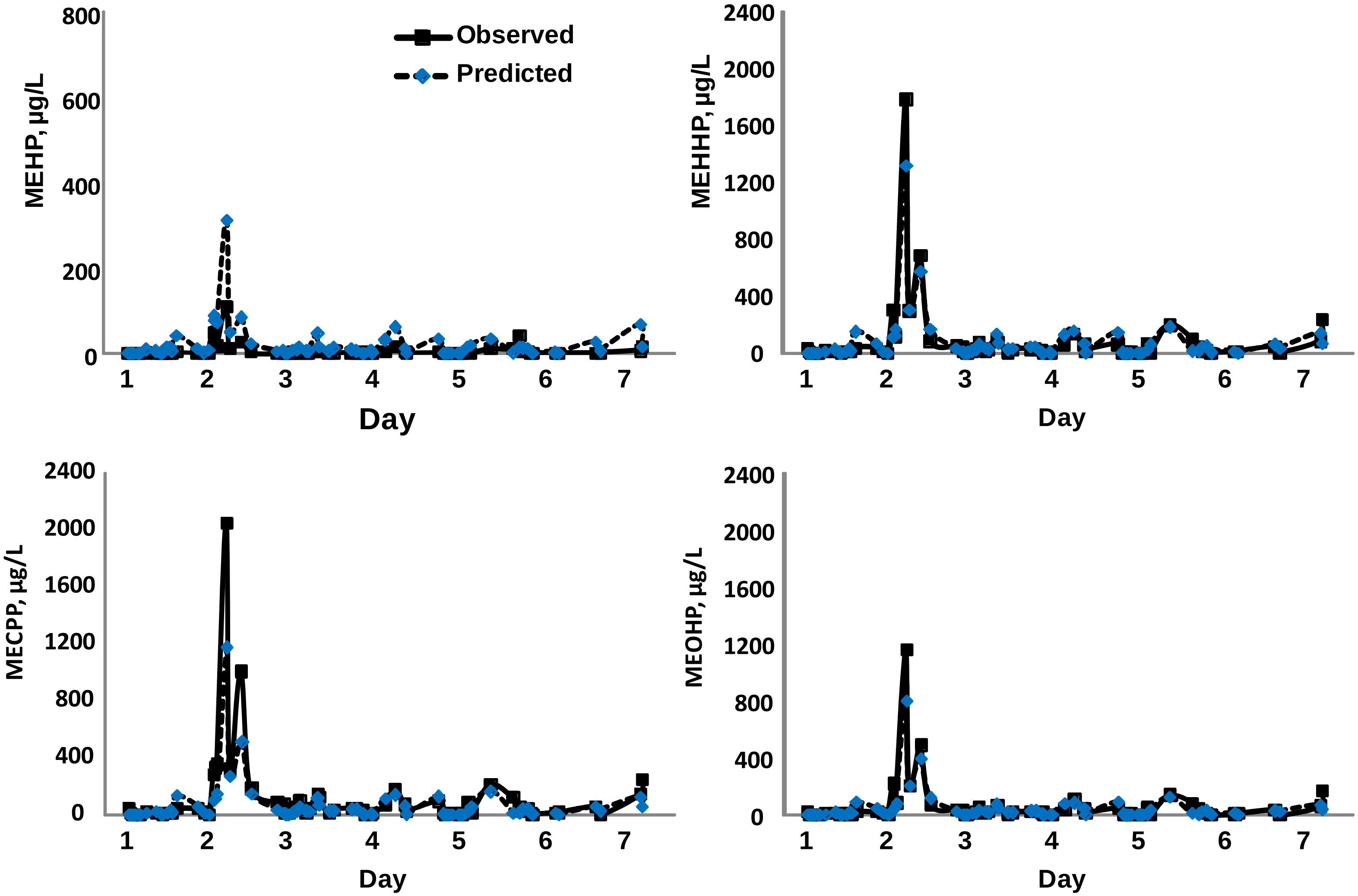

Figure S4. Observed and predicted urinary concentrations of the four DEHP metabolites, MEHP, MEHHP, MECPP, and MEOHP, for Subject 3.

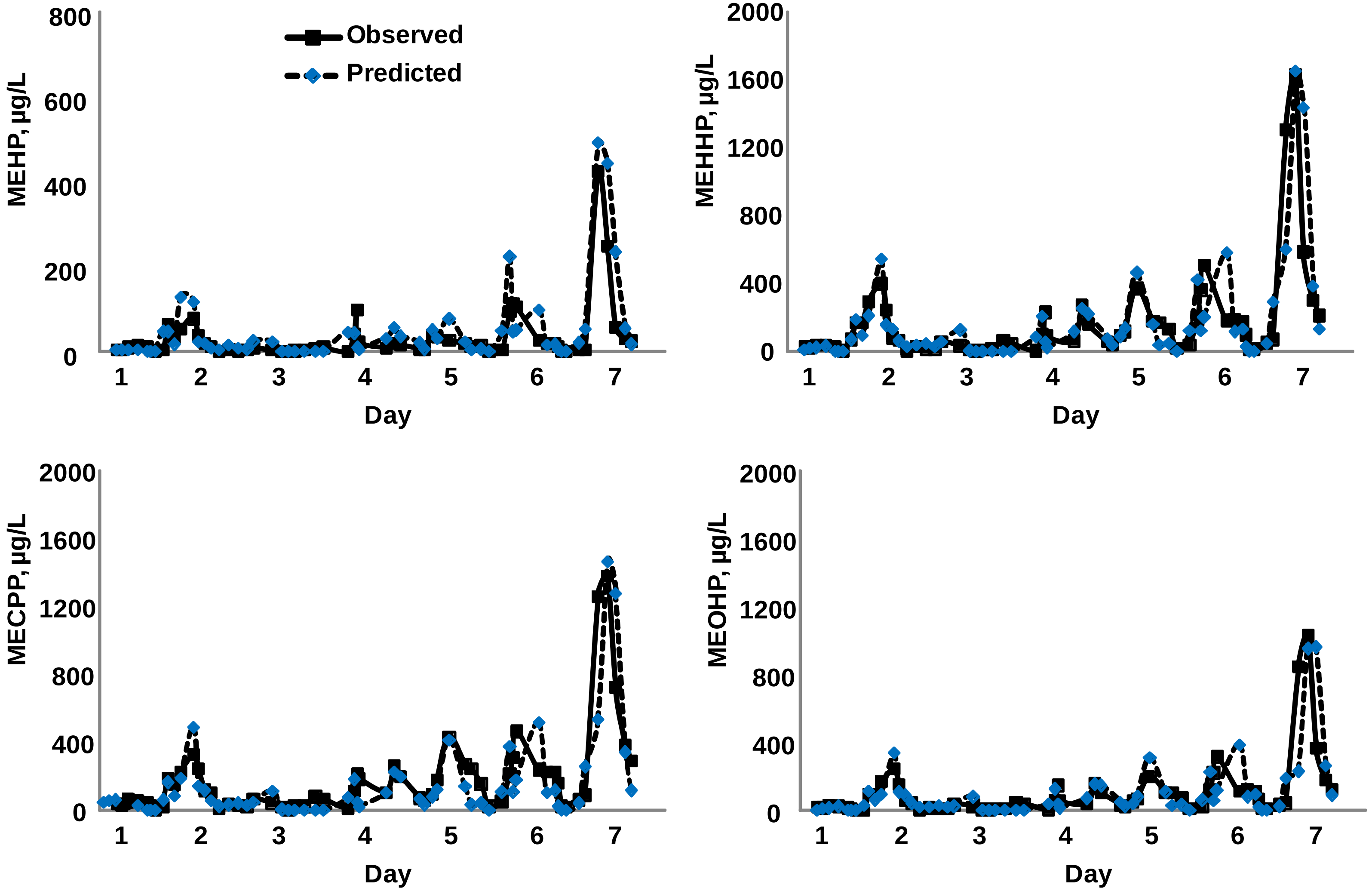

Figure S5. Observed and predicted urinary concentrations of the four DEHP metabolites, MEHP, MEHHP, MECPP, and MEOHP, for Subject 4.

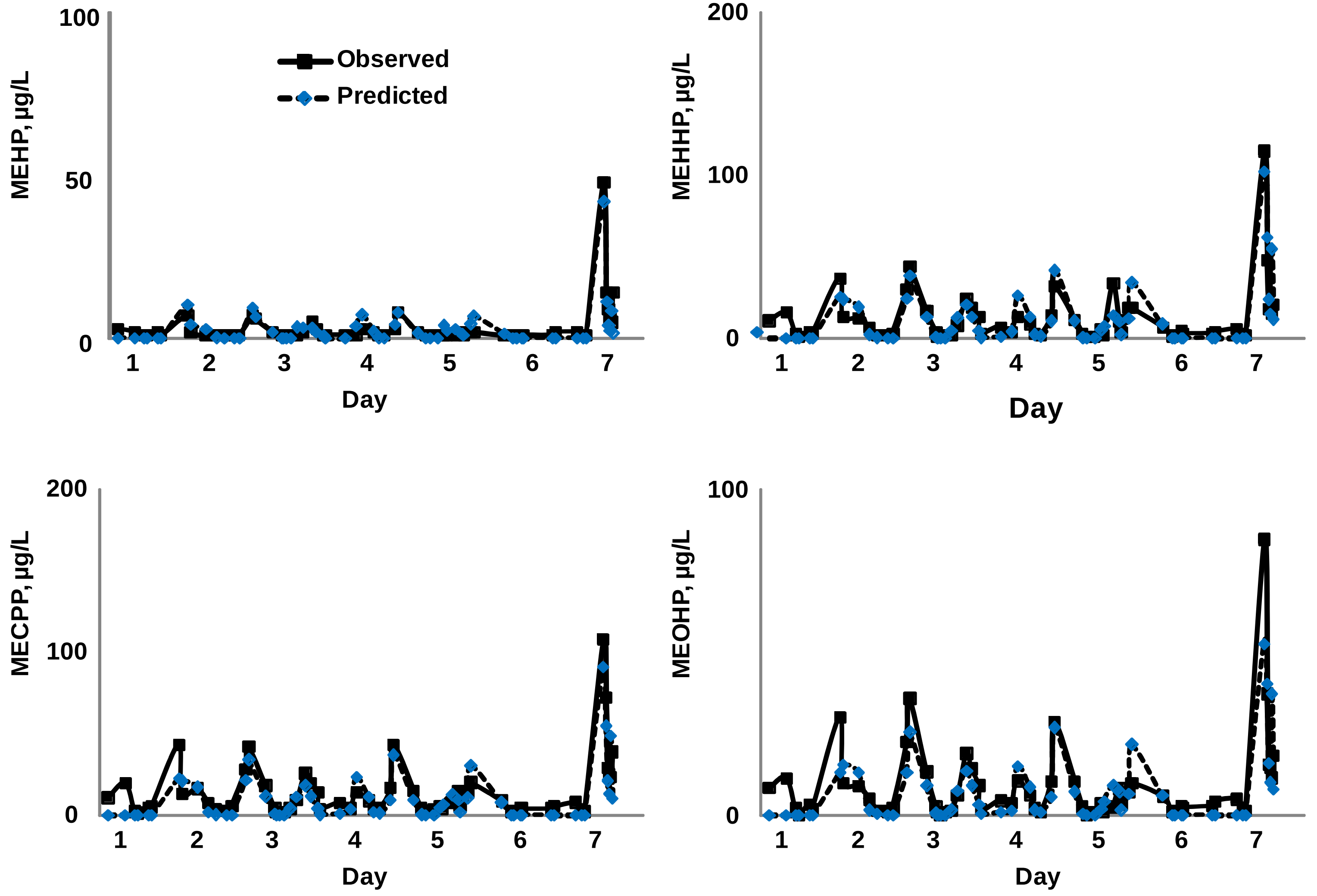

Figure S6. Observed and predicted urinary concentrations of the four DEHP metabolites, MEHP, MEHHP, MECPP, and MEOHP, for Subject 5.

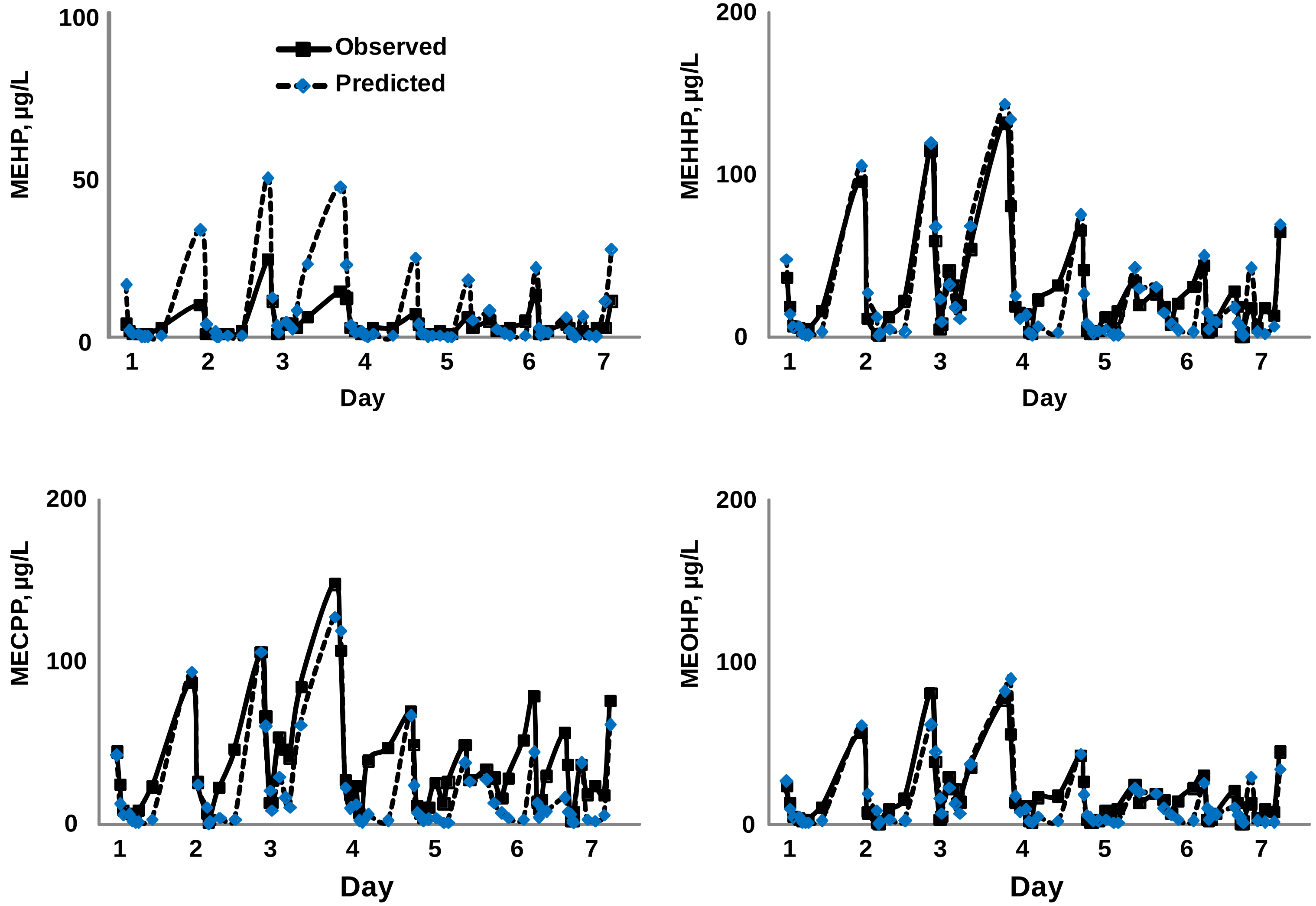

Figure S7. Observed and predicted urinary concentrations of the four DEHP metabolites, MEHP, MEHHP, MECPP, and MEOHP, for Subject 6.

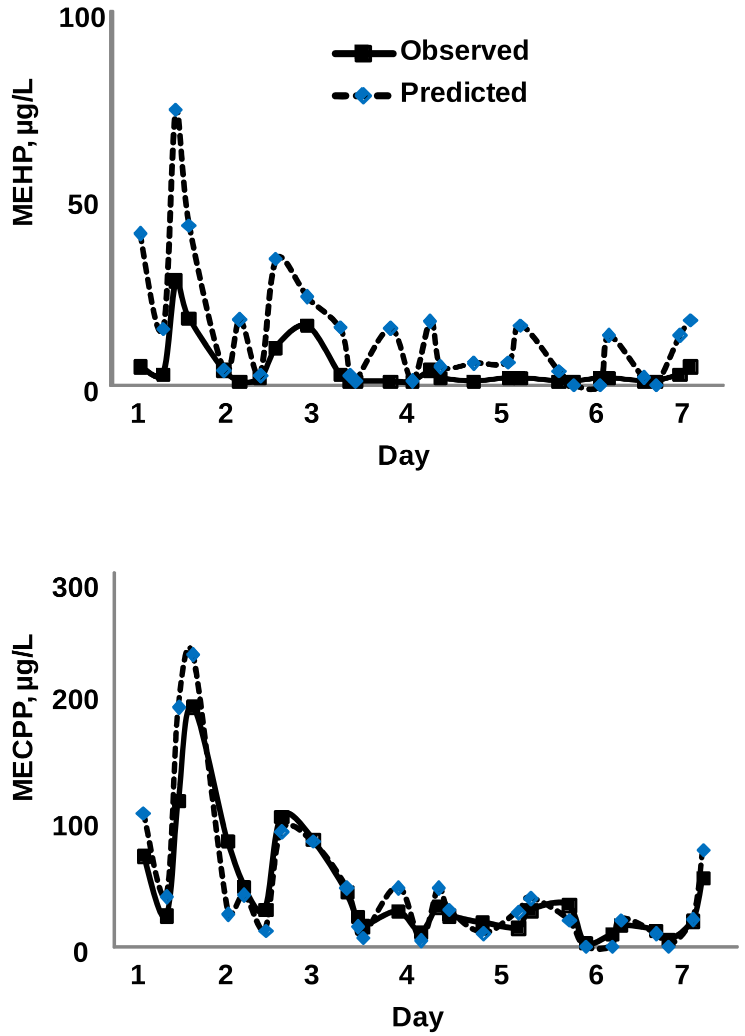

Figure S8. Observed and predicted urinary concentrations of the four DEHP metabolites, MEHP, MEHHP, MECPP, and MEOHP, for Subject 7.

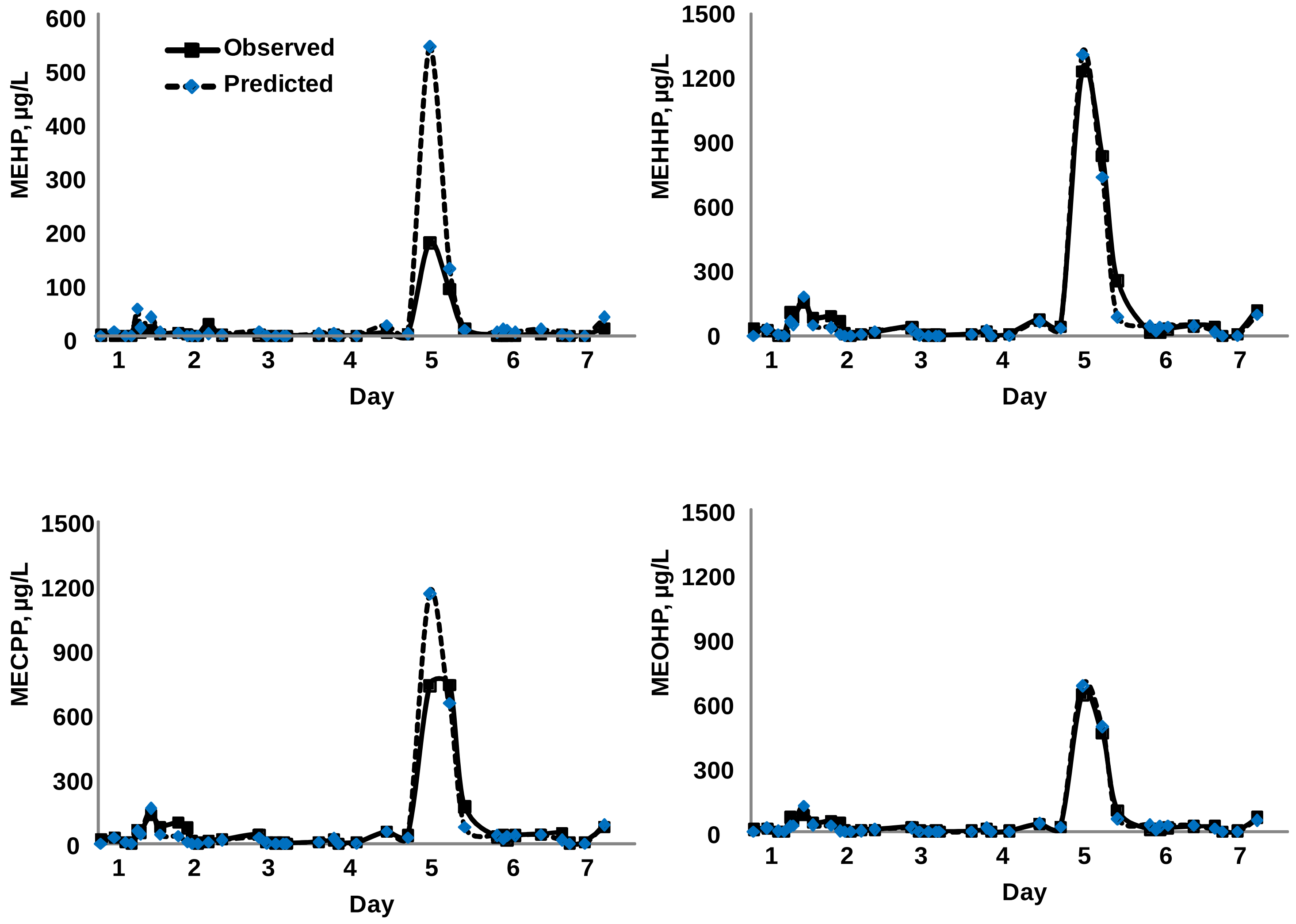

Figure S9. Observed and predicted urinary concentrations of the four DEHP metabolites, MEHP, MEHHP, MECPP, and MEOHP, for Subject 8.

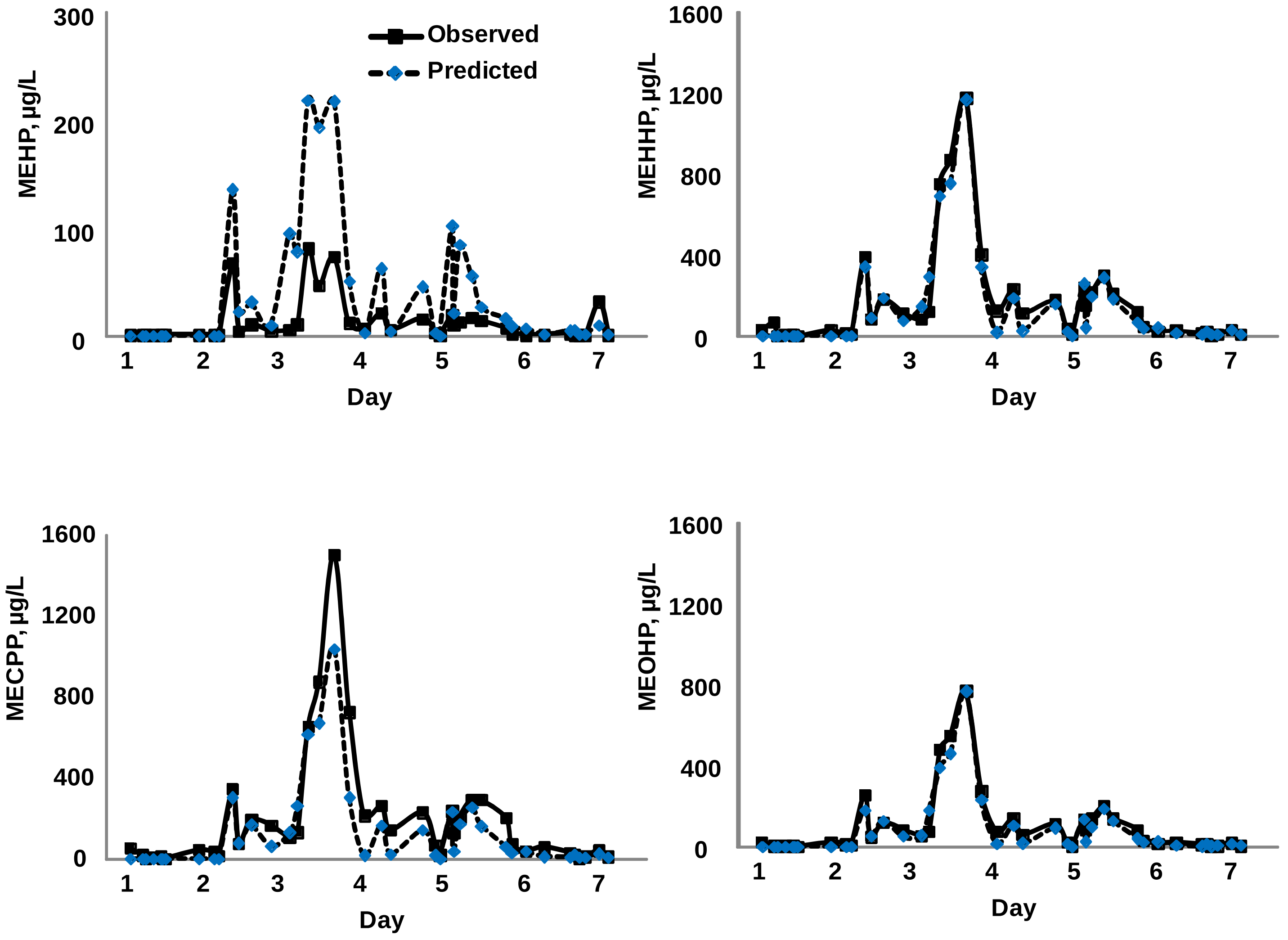

Supplement: (5.1 MB) PDF [file ehp.1205182.s001.pdf]
